# Anthropocene-related disease: The inevitable outcome of progressive niche modification?

**DOI:** 10.1093/emph/eoaa042

**Published:** 2020-11-10

**Authors:** Peter D Gluckman, Felicia M Low, Mark A Hanson

**Affiliations:** e1 University of Auckland, Koi Tū: The Centre for Informed Futures, Private Bag 92019, Auckland 1142, New Zealand; e2 University of Southampton, University Road, Southampton SO17 1BJ, UK

## Abstract

While the Anthropocene is often discussed in terms of the health of the planet, there has been less attention paid to its impact on the health of humans. We argue that there is now sufficient evidence of broad and growing adverse effects on human health to consider Anthropocene-related diseases and their impact on public health as a category of conditions needing specific recognition and preventative action. Using the examples of climate change-related health challenges, non-communicable disease, antimicrobial resistance and the unique challenges of the digital environment, we discuss how the profound and pervasive environmental changes of the Anthropocene can affect our health, with broad effects on societal health. We frame this concept in terms of human evolutionary history and cultural evolution’s runaway characteristics, reflecting our drive for continual and cumulative innovation for reasons beyond simply survival and Darwinian fitness. As the causative agents are often remote from those populations most adversely affected, prevention and mitigation require collective societal and policy actions.

**Lay summary:** There is increasing evidence that our uniquely evolved ability to modify our environments rapidly and at an accelerating pace is having impacts on our health, particularly non-communicable diseases and poor mental wellbeing. Reframing these public health challenges as Anthropocene-related diseases emphasizes the need for collective responsibility and systems approaches to prevention.

## INTRODUCTION 

The concept of the Anthropocene is widely accepted as an epoch of the planet’s history where human technological and related innovations have had significant and enduring impact on its physical and biotic environments [1, 2]. While the time when such impacts became observable is debatable, the dramatic increase in the planetary effects of human activity is clearly evident from the latter half of the last century [3]. This escalating rate of environmental change is too rapid for the operation of adaptive evolutionary processes in most species, particularly those with a long generation time, since such processes depend on selection of genotypes that confer fitness advantages. The impact of the Anthropocene is often discussed in relation to planetary health and the possible reduction in biodiversity from the extinction of a range of other species. However, discussion on how the Anthropocene impacts on the human condition and in particular on human health has largely focused on the direct and acute effects of climate change on health, for example in terms of extreme weather events or changes in the distribution of pathogen vectors [4, 5], rather than considering broader aspects.

In this article, we use the term Anthropocene in its broadly accepted sense, encompassing the modern-day impacts of the entire range of human activities. We do this to provide a framework by which to link concepts of cultural evolution and its effects on human biology and illustrate the framework by bringing together two related arguments. First, we propose that the timescale and the accelerating challenge of the Anthropocene is a result of our uniquely evolved niche modifying characteristics [6], which arise from our evolved biology and capacity for cumulative cultural evolution. Such evolutionary processes operate in our species for reasons that extend beyond the classical features of Darwinian fitness (i.e. survival and reproduction). Second, we argue that—in addition to direct, acute effects—there is now growing evidence of adverse effects of the Anthropocene on wider aspects of human health, in particular non-communicable diseases (NCDs) and mental health. This suggests that an emerging conflict between our biological and cultural evolution must be considered as a pathway to disease of growing concern. We propose that Anthropocene-related diseases and the public health challenges they pose should be specifically recognized as a category of conditions needing specific action. Importantly, such a framing would extend the scope of responsibility beyond the individual to collective responsibility. We argue that such reframing emphasizes the need to more broadly adopt systems approaches and policy actions to improve population health through pre-emptive interventions.

## HUMANS AS NICHE MODIFIERS

Evolutionary theory is based on the outcome of the interplay between changing environments and the biology of organisms that inhabit them. Some species have evolved specific traits or behaviors that buffer environmental change and thus sustain their Darwinian fitness by reducing the influence of such change [7, 8]. There are several distinct strategies by which they do this, broadly including adaptive physiology, adaptive developmental plasticity including polyphenism [7, 9], migration and niche construction where the species constructs a niche to buffer it from environmental variation [10]. The best known examples in animals of the latter are dam construction in rivers by the beaver, and the mounds built by termites to cope with large circadian variations in temperature.

Extending such concepts to humans requires additional considerations. Our species’ unique innovative capacities have been enabled by our evolved cognitive, sophisticated learning, communication and manual dexterity skills, as well as our abilities to store collective knowledge accumulatively. As the pace of our technological innovations has increased, we have increasingly modified the wide range of environments that we inhabit. But rather than being driven only by the need to sustain Darwinian fitness, our continual niche modifications have increasingly been fuelled by our desire to improve our quality of life, prosperity, intellectual and emotional stimulation and to support our social structure [6]. Thus, we would be best classified as niche *modifiers* [6]. However, as we will argue below, when extended beyond purely adaptive purposes, such innovative capacities have the potential to lead to unintended adverse consequences for our species: there is increasing evidence of such negative impacts now affecting our health [11].

Biotic effects have played important roles in our planetary evolution. Most notably, cyanobacteria are thought to be responsible for the ‘great oxygen event’ that led to massive rises in atmospheric oxygen and perhaps planetary cooling about 2.5 billion years ago [12]. However, such biotic influences are not comparable to what is now occurring. The environmental, biological and social changes of the Anthropocene are large, relentless, rapid and pervasive.

Humans have likely modified their environments since their first origins, as did their hominid ancestors. From a broader environmental point of view, however, the effects of human activity become clearly evident from the times of the agricultural revolution and classical urbanization, growing during the Middle Ages and then increasing substantially at the time of the Industrial Revolution [13]. Indeed, the increase in rice farming during the Bronze Age has been linked to the increase in atmospheric methane concentrations beginning about 5000 years ago [14]. As the global population has increased rapidly as a result of technological progress and has been associated with human and economic development in nearly all societies, especially from the mid-20th century, the magnitude of human impacts has accelerated exponentially and produced cumulative effects [3]. The consequences raise critical concerns about our ability to cope with the challenges we are continuing to impose on ourselves as a result of our innovations [11].

A fundamental feature of our socio-technological evolution is that we continually modify our environment. This is in contrast to niche constructing species or migrating species that each, in different ways, generate an effective equilibrium between their phenotype and their niche [8]. In the case of niche constructing species, the niche constructing behavior is inherited in a stable way, such that the niche does not change over many generations. The concept of niche modification describes behaviors where the environmental modifications are progressive and equilibrium is not reached [6]. In recent times, humans have increasingly not been in equilibrium with their environment (broadly defined), because cultural evolution has conferred a different purpose on these modifications, which are now related broadly to our perceptions of our quality of life rather than sustenance of Darwinian fitness. This has not only positive consequences—which is why they persist through cultural selection—but also negative effects. To partially address these negative consequences, we have often resorted to new innovations to counter the detrimental effects of previous ones. However, this may not always be possible, resulting in persistent and deleterious impacts on the human condition. We will explore this idea through four health challenges posed in the contemporary Anthropocene, in order to consider whether this strategy may suggest sustainable solutions or whether other approaches are needed.

## ACUTE AND CHRONIC HEALTH CHALLENGES ARISING FROM THE ANTHROPOCENE

The development of fossil fuel technologies underpinned the Industrial Revolution but has led in relatively short order to rapid climate change and its attendant impacts on many aspects of our lives, including our health [15]. Warmer temperatures are forecast to extend the geographical distribution of insect vector-borne diseases such as malaria and dengue fever [16]. They have also increased the risk of forest and bush fires, which have impact on air quality and damaged property even in urban regions [17]. This has caused not only worsened respiratory health especially among asthma sufferers [18] but also death in vulnerable members of the population from heat exhaustion and dehydration [19]. There is also concern that the frequency and severity of events such as hurricanes and extreme rainfall is increasing as a result of climate change. Rising sea levels pose threats to food security, sanitation and medical facilities in coastal communities, with flooding having the potential to devastate dwellings, crops and infrastructure [20, 21]. The combination of rising heat and humidity will make the risk of heat stroke a real challenge in some regions [22]. Regrettably, the populations most affected by such challenges are often those in low-resource settings that have benefited the least from the innovations that led to such threats in the first place [23]. Vast human migrations seem likely as sea levels rise and some regions become uninhabitable.

It is becoming increasingly appreciated that an even greater concern than the immediate damage to property and physical health may be the impact of environmental disaster on mental health in many members of the population, whether it be because of displacement, loss of livelihood or anxiety about the future [24]. Eco-anxiety is already a major issue for many young people [25]. Such challenges to mental wellbeing will not be quickly resolved and will incur major social and financial costs.

This is also true of the second major challenge arising in the contemporary Anthropocene: NCDs, which account for 63% of deaths globally [26], with 86% of premature NCD-related deaths occurring in low-middle-income countries [27]. These diseases, primarily cardiovascular and respiratory disease, diabetes and some cancers, develop slowly across the life-course; once developed they can be treated, often at great cost and in technologically intensive ways, but not easily cured. As such, they carry a very substantial social and economic burden. These conditions are often labelled ‘lifestyle’ diseases given their links to modern obesogenic environments resulting from innovations in food production, preservation and transport. However, that term implies individual choice and responsibility for selecting a given lifestyle—a sentiment that is reflected in many policy positions across the world, with a reluctance of governments to play their role in addressing the underlying systems [28]. The term also gives little weight to the role of early development and parental effects in influencing NCD risk, despite the abundance of robust epidemiological, clinical and experimental evidence [29]. There is currently no country in the world in which the challenge of obesity is being met [30], and the situation is made even more worrying by the high prevalence of obesity in young adults of reproductive age, and the growing evidence that risk of overweight and obesity can be biologically transmitted across generations [31]. Because such inheritance of risk does not easily lend itself to intervention by behavioral choices, and because the wider social determinants of risk which are largely outside individual control are increasingly recognized [32], we suggest that obesity and NCDs should be considered to be Anthropocene-related rather than the misnomer of ‘lifestyle’ diseases. The latter framing obscures needed remedial actions.

A further health challenge stemming from Anthropocene-related human innovative behaviors is antibiotic resistance. The industrial scale manufacture and use of antibiotics provided a dramatic breakthrough in combating bacterial infections in humans and could be seen as fitness-enhancing. However, as a result of the extension of their usage to promote growth in intensively farmed animals, as well as injudicious use in humans, we are now engaged in a biological arms race between the development of new antimicrobial agents and the corresponding emergence of resistant strains of pathogens [33, 34]. This is especially worrying as the threat of epidemics is increased by rapid and widespread long-distance travel [35]. While viral in origin, the COVID-19 pandemic highlights the risks we face in growingly interconnected world.

## THE DIGITAL ENVIRONMENT AND ITS UNIQUE CHALLENGES

As our fourth and perhaps most challenging example, we suggest that digital technologies and the digital society in which we now live pose arguably the most pervasive health risks arising from our cultural evolution [11]. Since the invention of the internet, the ‘virtual’, digital world has become an essential part of the human environment. The internet was initially conceived as being a scientific tool, but the resulting digital technologies have now been adopted very rapidly. In 2019, an estimated 54% of the global population, or 4.1 billion people, were internet users [36]. Until recently, discussions about the impact of digital technologies have mostly centered on their largely positive impact on productivity, the economy, and as a means of empowering people through a greater ability to communicate and to access information [37, 38]. However, despite recognized positive effects such as the greater democratization of knowledge, it is increasingly evident that the digital transformation is having potential negative effects on mental wellbeing of individuals in many contexts. The psychological consequences can lead to behavioral changes which can have knock-on effects, extending far beyond the individual to wider society [39–41]. Well-known examples concern radicalization and extreme political views leading to terrorist attacks and the manipulation of information to affect societal behavior and beliefs.

A part of this downside of the digital environment can be attributed to social media use, which is now a fundamental, ubiquitous aspect of the global human environment; users of just Facebook alone account for one in three people in world, or more than two-thirds of internet users [42]. A large body of research is demonstrating how social media can perturb the structure of social interactions, and impact on cohesion, resilience and many aspects of wellbeing [39]. Examples include the impact of parental distraction by mobile devices on familial interactions [43], and through to the proliferation of disinformation leading to an ill-informed, politically polarized society [44]. Of particular concern is the growing evidence from psychological, psychiatric and neuroimaging studies suggesting an association between digital technology use and altered brain development and cognition, or poorer mental health, in children or young people [45–50]. This has important implications because perturbations in a child’s rapidly developing brain may impact on their longer-term health and wellbeing, while youth mental health morbidity—besides being of obvious public health concern—further poses a challenge to societal resilience. Thus, even relatively small effect sizes may have far-reaching consequences.

From an evolutionary point of view, the human brain evolved to operate within relatively small social networks [51], and so may have limited capacity to cope with dramatic expansions of social groups, particularly in the virtual world. Furthermore, the rapid spread of misinformation through large networks can directly lead to harmful health outcomes. For example, the anti-vaccination movement, which has led to the recent measles epidemic around the globe [52] and may well impact on the uptake of COVID-19 vaccines, shows how the manipulation of social media by a small group of individuals with extreme views can undermine the adoption of a highly effective innovation (immunization) by a much larger population, with loss of herd immunity potentially affecting the lives of many individuals remotely. Recent research has examined how online groups expressing extreme views form, the processes underlying their resilience and how they may be countered [53].

While some now evolving digital technologies such as artificial intelligence may offer advantage to many, there is potential for greater numbers of people to experience disadvantage [54], and this may exacerbate existing inequalities or significantly alter social structures and the sense of safety and security [55, 56]. At a broader societal level, citizens’ rights to privacy, agency and autonomy are seemingly becoming eroded—not just in non-democratic societies, but even so-called liberal democracies as well [57, 58]. These issues, together with the proliferation of online misinformation and disinformation, present a threat to social cohesion and societal resilience. Furthermore, as a commercial phenomenon, digital technology innovations have concentrated wealth and power in the hands of a few individuals. Overall, their indirect effects may potentially be even more widespread than those of climate change or highly processed foods. These concerns are amplified when considering that we are likely to be merely at the beginning of innovative digital modifications to our environment, and they raise questions about the effects on our mental health arising from such evolutionarily novel expanded, rapidly changing and extremely hierarchical social structures.

## THE CHALLENGES OF TACKLING ANTHROPOCENE-RELATED DISEASES

We use our evolved ingenuity and our ability to engage continually in niche modification to develop new technological solutions to the problems caused by existing ones. Yet, the impact of our innovations on our lifestyle and expectations is such that there is seldom any acceptable way to easily return to the situation which existed before the effects of technologies were appreciated [11]. Thus, despite the emergence of many unforeseen impacts of the internet and its associated technologies on wellbeing at individual, societal and transnational levels, it would be unthinkable to revert to an internet-free world. Likewise, despite Anthropocene-associated climate change and the development of new technologies to reduce greenhouse gas emissions from energy production, transport or agriculture, it is difficult to conceive of living without any fossil fuel use or without food production on an industrial scale. Instead, we are applying our ingenuity to develop new geo-engineering solutions such as reflecting earthbound solar radiation and carbon dioxide capture [59]; however, none of these has been trialed in the real world on a large-scale, and they may well have unintended effects on forest life and biodiversity [60]. In the same vein, the problem of the spread of malaria has prompted the development of meiotic gene drive technologies to affect the reproductive capacity of mosquitoes, which may themselves have other, as yet unforeseen consequences [61].

Similar considerations apply to the Anthropocene-related diseases and health issues discussed earlier. To deal with the adverse effects of ultra-processed foods and sedentary lifestyles leading to obesity, we have resorted to new strategies, ranging from appetite-modifying drugs and bariatric surgery to mobile apps aimed at modifying dietary behavior, largely ineffectively. Assisted reproductive technologies are sometimes necessary to counter the adverse effects of obesity on fertility. While some movements have attempted to adopt aspects of what were perceived as lifestyles of our evolutionary past (e.g. the so-called ‘Paleo’ diet), they do not represent a collective behavioral solution. Once again, the health impact of the obesogenic environment is disproportionately greater in low- and middle-income countries [62], most dramatically in small island developing states where traditional diets have been largely replaced by ‘Western’ diets in a rapid recent transition.

The challenge of antimicrobial resistance is similarly demanding the development of new technologies in the form of novel drugs to combat resistant strains of bacteria. We will be at the losing end of this arms race if a strain of an existing pathogen evolves for which there is no effective available drug and/or a vaccine has not been developed [63]. This scenario is posed by methicillin-resistant *Staphylococcus aureus* (MRSA), the so-called ‘superbug’ that is resistant to multiple commonly used antibiotics and is currently treated with last-resort medications such as vancomycin. MRSA strains both partially resistant and fully resistant to vancomycin have already emerged, with dire clinical outcomes especially for infections involving the latter [64].

Many of these considerations have been brought into sharp focus by the COVID-19 pandemic—itself an exemplar of the greater risks of novel zoonoses as populations expand and technological advances in travel promote rapid global spread.

## OUR FUTURE IN THE ANTHROPOCENE

The examples discussed above strongly favor a diagnosis of the effects of the Anthropocene on our health which are evident now and are likely to be even more prevalent in the near future. But how far are we from being able to propose solutions?

To do so, we argue that there is first a critical need to reflect on the broader impact of the Anthropocene on our biology and health as individuals and societies [6, 11]. This also raises questions about whether there are limits to our ability to modify our environment for our long-term physical and mental health. Addressing such questions requires systems thinking rather than a narrow, reductionist approach, because the challenges posed by the Anthropocene raise complex, multi-dimensional social issues to which there is no simple solution [65]. At the same time, we note that systemic changes are generally much more difficult to implement than changes at the individual level. For example, food availability in developing countries is dependent on multiple interrelated factors for which there are likely to be lags in effects observed should interventions be applied. Thus, efforts to improve local public health policy may be rendered ineffective in the context of a globalized economy.

What is evident, however, is that there will not be a one-size-fits-all solution to the challenge of Anthropocene-related diseases. Indeed, causation and prevention cannot simply be matters of individual ‘lifestyle’ choices, but instead require collective and policy actions. Societies and policymakers need to have critical conversations about the limits and uses of evolving technologies. This helps distinguish short-term direct benefits or risks from longer-term collective benefits or costs. A clear failure to do so—reflective of individualistic choices—can be seen in our slow progress in mitigating climate change.

Few countries currently have effective domestic systems for integrating technological and scientific thinking and foresight into their policymaking processes. It is important to recognize the areas where rapid technological innovations may have adverse effects on us, even if we envisage developing further technologies to ameliorate such effects. As is so often the case in areas of public health, how the problem is framed may be important to our success in addressing it.

Given our species’ unstoppable and evolved capacity for niche modification, further rapid changes in our environment, driven by our innovative technological capacities, are inevitable. But while technologies bring many benefits, they will not be without significant impacts at every level, from the individual to organized society. We will need to reflect on whether new ways of evaluating potentially disruptive technologies are required. This is made more complex in that technologies now often arise from the private sector with little warning and quickly span jurisdictions. Governments may be reluctant to pre-emptively regulate such developments in case this impedes innovation, yet by the time they become aware of the possible or actual downsides of a new technology, it is not readily possible to find regulatory solutions. A clear example of this is the live-streaming on Facebook of the March 2019 Christchurch, New Zealand, mosque atrocity, and the subsequent limitations encountered in preventing its online distribution on multiple other platforms [66].

With respect to our digital society, virtually no country has adopted a holistic approach to analyzing the digital transformation and the multitude of non-technical issues that may arise. Yet we need to consider how to instill greater psychological resilience in individuals, and social resilience in communities, to meet the rapid and seemingly inevitable changes affecting our health. Broader definitions of wellbeing that take into account self-perceived connectedness and other social determinants of health may be needed [39].

Humans are unique among all species in their capacities for rapid and cumulative cultural evolution, but now our trajectory is one of runaway cultural evolution for which we are ill-prepared. The issues of whether society has the appropriate institutions to deal with the complex, rapidly moving technological developments that operate across jurisdictional borders, and their possible effects on our health, need greater consideration by ethicists, political scientists, and policy makers, both domestically and globally.

**Conflict of interest:** None declared. 
